# Maternal vitamin D depletion alters DNA methylation at imprinted loci in multiple generations

**DOI:** 10.1186/s13148-016-0276-4

**Published:** 2016-10-12

**Authors:** Jing Xue, Sarah A. Schoenrock, William Valdar, Lisa M. Tarantino, Folami Y. Ideraabdullah

**Affiliations:** 1 Nutrition Research Institute, University of North Carolina, Kannapolis, NC 28081 USA; 2 Department of Genetics, School of Medicine, University of North Carolina, Chapel Hill, NC 27599 USA; 3 Neurobiology Curriculum, University of North Carolina, Chapel Hill, NC 27599 USA; 4 Lineberger Comprehensive Cancer Center, University of North Carolina, Chapel Hill, NC 27599 USA; 5 Division of Pharmacotherapy and Experimental Therapeutics, Eshelman School of Pharmacy, University of North Carolina, Chapel Hill, NC 27599 USA; 6 Department of Nutrition, Gillings School of Public Health, University of North Carolina, Chapel Hill, NC 27599 USA

**Keywords:** Epigenetic inheritance, Parent of origin, Vitamin D, Maternal diet, Imprinting, Collaborative Cross

## Abstract

**Background:**

Environmental perturbation of epigenetic mechanisms is linked to a growing number of diseases. Characterizing the role environmental factors play in modifying the epigenome is important for disease etiology. Vitamin D is an essential nutrient affecting brain, bone, heart, immune and reproductive health. Vitamin D insufficiency is a global issue, and the role in maternal and child health remains under investigation.

**Methods:**

We used Collaborative Cross (CC) inbred mice to characterize the effect of maternal vitamin D depletion on offspring phenotypic and epigenetic outcomes at imprinted domains (*H19/Igf2*, *Snrpn*, *Dlk1/Gtl2*, and *Grb10*) in the soma (liver) and germline (sperm). We assessed outcomes in two generations of offspring to determine heritability. We used reciprocal crosses between lines CC001/Unc and CC011/Unc to investigate parent of origin effects.

**Results:**

Maternal vitamin D deficiency led to altered body weight and DNA methylation in two generations of offspring. Loci assayed in adult liver and sperm were mostly hypomethylated, but changes were few and small in effect size (<7 % difference on average). There was no change in total expression of genes adjacent to methylation changes in neonatal liver. Methylation changes were cell type specific such that changes at *IG-DMR* were present in sperm but not in liver. Some methylation changes were distinct between generations such that methylation changes at the *H19ICR* in second-generation liver were not present in first-generation sperm or liver. Interestingly, some diet-dependent changes in body weight and methylation were seemingly influenced by parent of origin such that reciprocal crosses exhibited inverse effects.

**Conclusions:**

These findings demonstrate that maternal vitamin D status plays a role in determining DNA methylation state in the germline and soma. Detection of methylation changes in the unexposed second-generation demonstrates that maternal vitamin D depletion can have long-term effects on the epigenome of subsequent generations. Differences in vitamin D-dependent epigenetic state between cell types and generations indicate perturbation of the epigenetic landscape rather than a targeted, locus-specific effect. While the biological importance of these subtle changes remains unclear, they warrant an investigation of epigenome-wide effects of maternal vitamin D depletion.

**Electronic supplementary material:**

The online version of this article (doi:10.1186/s13148-016-0276-4) contains supplementary material, which is available to authorized users.

## Background

In mammals, maternal diet plays an important role in offspring early development from conception through perinatal stages. During gestation, the embryo undergoes extensive de novo tissue and organ development requiring rapid cell divisions and differentiation. Development continues postnatally to generate a fully functioning organism. An essential part of these developmental stages is epigenetic reprogramming. Parental germline epigenetic profiles are erased after fertilization and reestablished in the offspring’s developing germline and soma. In this way, epigenetic profiles are reset in each generation based on cell fate. These events are spatially and temporally regulated [1, 2]. Epigenetic programs established during early development are required to regulate transcriptional and metabolic processes occurring pre- and postnatally including those required for long-term health [3–5]. Thus, maternal diet-dependent changes in epigenetic programming during early development have the potential to alter offspring short- and long-term health [6].

Vitamin D is an essential micronutrient that plays an important role in many developmental processes including brain, bone, heart, immune, and reproductive health [7–11]. Dietary vitamin D is converted in the liver to the inactive metabolite 25-hydroxyvitamin D, 25(OH)D, before being converted in the kidney to the active metabolite 1,25(OH)_2_D. Endogenous production of 25(OH)D_3_ occurs via conversion of 7-dehydrocholesterol (DHCR7) to cholecalciferol by UV-B exposure in sunlight [12]. Vitamin D insufficiency is a global problem with recent reports as high as 50 % among children, adults, and pregnant women (reviewed by Holick et al. and Saraf et al. [13, 14]). Approximately 32 % of Americans are at risk for vitamin D inadequacy, having less than 25 ng/ml serum 25-hydroxyvitamin D [15]. Among females 12–44 years of age, 28 % of pregnant/lactating females and 37 % of non-pregnant/non-lactating females are at risk of vitamin D inadequacy [15]. The active vitamin D metabolite 1,25(OH)_2_D has both genomic and non-genomic functions. Primarily, 1,25(OH)_2_D functions by binding to the vitamin D receptor (VDR) and initiating gene expression changes via genomic VDR response elements. 1,25(OH)_2_D also binds to transmembrane or cytosol receptors to initiate downstream events (as reviewed by Blomberg Jensen [11]).

Very little is known about the role of vitamin D in epigenetic mechanisms. However, recent findings implicate vitamin D in regulating histone modifications [16, 17] and DNA methylation [18]. Pereira and colleagues previously reported differential regulation of lysine (K)-specific demethylases (KDMs, KDM2B/4A/4C/4D/5A/5B) in 1,25(OH)_2_D_3_ supplemented culture of SW480-ADH human colon cancer cells compared with unsupplemented cultures [16]. The KDM4 family targets histone H3 lysine 9 di-/tri-methylation (H3K9me2/3), modifications with critical roles in fetal reprogramming [19, 20]. A separate study by Padi et al. reported microRNA-627-dependent downregulation of the H3K9me1/2 demethylase, KDM3A, in 1,25(OH)_2_D_3_ supplemented cultures of several human colon cancer cells and in in vivo murine xenograft tumor models [21]. In support of this finding, 1,25(OH)_2_D_3_ treatment in cell culture was linked to increased enrichment of H3K9me2 and hypermethylation at target gene promoter regions [22]. In humans, Junge et al. reported significantly different DNA methylation profiles (508 regions changed) between newborns with high (>19.9 ng/ml) and low (<7.9 ng/ml) 25(OH)D in cord blood [18]. These findings implicate a role for vitamin D in determining epigenetic state and highlight the need for further study to determine the mechanism and potential links to health and disease.

To address the role of maternal vitamin D in determining offspring epigenetic state, we used a mouse model to characterize whether depletion of maternal vitamin D during gestation and lactation affects epigenetic and developmental outcomes of progeny. The outcomes measured to infer developmental differences were body weight, body composition, testes weight, and sperm count. The epigenetic outcomes measured were DNA methylation states at differentially methylated regions (DMRs) that regulate imprinted genes with key roles in pre- and postnatal growth or adiposity [23–26]. These included known imprinting control regions (ICRs). ICRs are unique compared to other DMRs, such that DNA methylation state is established at ICRs in the germline in a parent of origin-dependent manner then stably maintained post-fertilization in all cell types throughout life, regardless of changes in gene expression. Thus, compared with other epigenetically regulated loci, ICRs provide a more direct assessment of epigenetic inheritance [27–29]. Also, because of this “protected” state, ICRs are thought to be more resistant to epigenetic perturbation. We investigated both maternally and paternally methylated DMRs and compared methylation changes in offspring liver and sperm to characterize vitamin D-dependent regulation of epigenetic state in soma and germline over two generations.

To maximize the robustness of phenotypic response to vitamin D deficiency, we used Collaborative Cross (CC) inbred mouse strains (CC001/Unc and CC011/Unc [30]). CC lines are recombinant inbred strains generated from intercrossing eight founder lines (A/J, C57BL/6J, 129S1/SvImJ, NOD/ShiLtJ, NZO/HILtJ, CAST/EiJ, PWK/PhJ, and WSB/EiJ) in a way that reduces the artificially created genomic structure of traditional inbred lines, and better mimics the types, combinations, and genomic distributions of genetic information found among non-artificially selected populations [31]. These lines incorporate a majority of the genetic variation present among traditional laboratory inbred mouse strains and are known to exhibit more robust levels and novel types of phenotypic changes not observed in traditional laboratory inbred strains [32–34].

We utilized reciprocal crosses between CC001/Unc and CC011/Unc (cross 1: CC001 female × CC011 male and cross 2: CC011 female × CC001 male) to assess the presence of parent of origin effects on phenotypic and epigenetic response to vitamin D deficiency. Parent of origin (PO) effects are defined as molecular or phenotypic outcomes that are dependent on maternal vs. paternal origins of the genome [35]. These can be influenced by genetic (e.g., DNA sequence differences), maternal differences (e.g., uterine effects), and epigenetic differences (e.g., imprinted genes). PO effects have been widely demonstrated to influence complex traits; however, the role in dietary responses is limited [36]. Here, we assess (1) overall dietary effects (diet, maternal, or grandmaternal vitamin D-deficient diet compared to control diet); (2) diet-independent parent/grandparent of origin effects (PO/GPO, cross 1 compared to cross 2, samples from different diets combined); and (3) diet-dependent parent/grandparent of origin effects (diet × PO/GPO, both diet and cross differences assessed in an interactive model).

We found that depletion of maternal vitamin D results in perturbation of DNA methylation state at ICRs in somatic cells and to a lesser degree in germ cells in two generations of offspring. When maternal exposure affects both the first-generation (G_1_) offspring and unexposed second-generation (G_2_) offspring, it is known as a multigenerational effect with the assumption that dietary effects in the unexposed G_2_ are due to inheritance of perturbation occurring in the G_1_ [37, 38]. We also observed multigenerational diet-dependent effects on body weight and body composition while testes weight was only affected in the first generation. Interestingly, the extent and direction of many of the dietary effects was dependent on the direction of the reciprocal cross (CC001 × CC011 vs. CC011 × CC001), thus demonstrating a parent of origin effect. The influence of grandmaternal vitamin D status on phenotypic and epigenetic outcomes demonstrates inheritance of perturbed epigenetic landscapes. However, the mechanism of transmission is indirect since for the most part, the same loci were not affected between generations. Thus, maternal vitamin D depletion most likely causes heritable disruption of global epigenetic programming in the offspring.

## Results

### Dietary depletion of circulating plasma 25(OH)D is dependent on genetic background

**Fig. 1:**
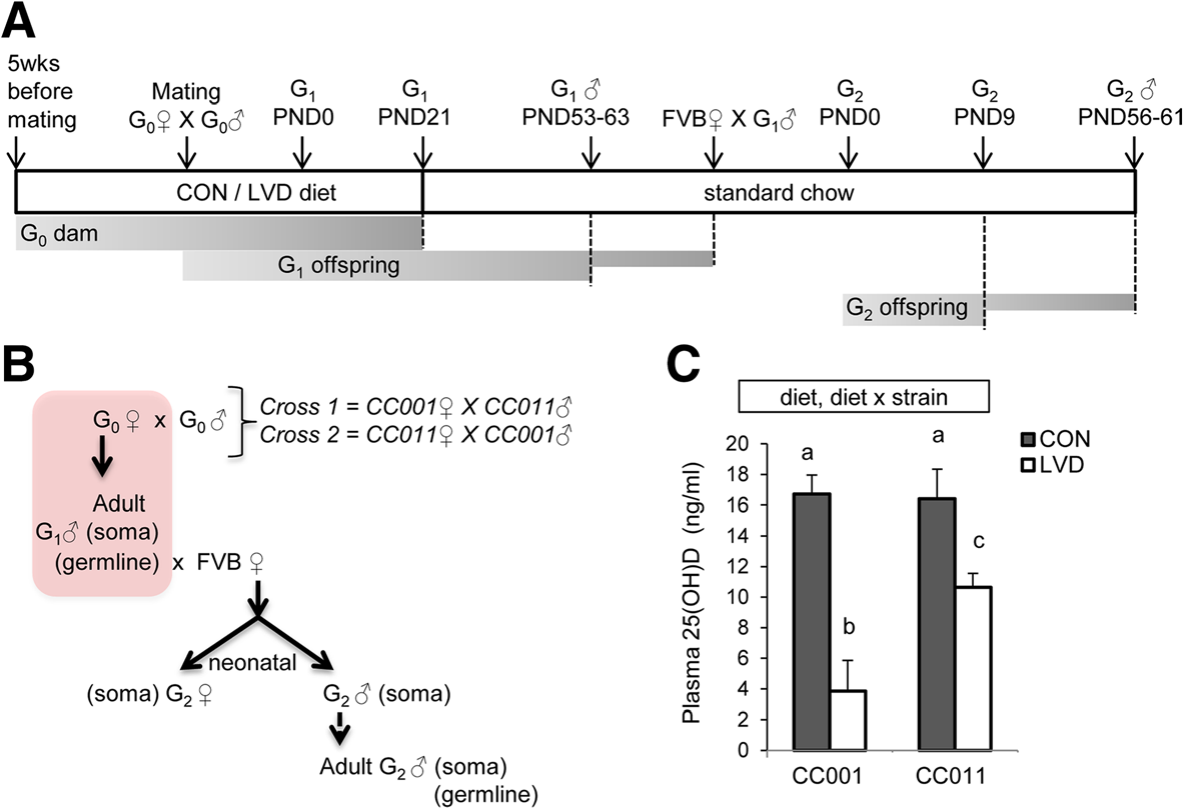
Experimental crosses and dietary treatment scheme. **a** Dietary treatments are indicated in *middle block*: CON (AIN-93G), LVD (lacking vitamin D), standard chow (Teklad 8604). *Arrows* above indicate treatment at developmental timepoints. *Shaded regions* below dietary treatment scheme indicate treatment windows and time of harvest (*dashed vertical line*). **b** Illustration of crosses used in paternal transmission of genomes exposed to vitamin D depletion (*highlighted region* depicts direct exposure), developmental timepoints and cell types tested (*parentheses*). **c** Maternal (G_0_ dam) plasma 25(OH)D levels (*N* = 5, 3, 5, 6, in order from *left* to *right*). *X*-axis indicates dam strain. *G*_*0*_ parental generation 0, *G*_*1*_ first generation of offspring, *G*_*2*_ second generation of offspring, *PND* postnatal day. *Different letters* on bars represent statistically significant groups with *p* value <0.05. *Panel above the graph* lists all statistically significant (*p* value <0.05) comparisons determined by regression analysis for overall diet-dependent effects (diet) and diet × strain interactive effects (diet × strain)

To determine the extent of vitamin D depletion in G_0_ dams fed diet lacking vitamin D (LVD) compared with dams on control diet (CON) (Fig. 1a), we compared circulating plasma 25(OH)D levels [39] of dams at pup weaning. LVD dams had significantly lower levels of plasma 25(OH)D compared with CON dams (*p* = 1.98 × 10^−5^, Fig. 1c). Depletion was not complete despite being on diet lacking vitamin D for more than 11 weeks. This may be due to the limited exposure to UVB light in the vivarium during dietary treatment (see the “Methods” section) or adipose storage release. Interestingly, the extent of 25(OH)D depletion in CC001 dams was significantly greater than in CC011 dams (*p* = 0.034, Fig. 1c) demonstrating a genetic influence on response to dietary vitamin D depletion.

### Maternal vitamin D depletion alters G_1_ and G_2_ offspring developmental outcomes in a parent and grandparent of origin-dependent manner

We next assessed whether maternal vitamin D depletion affects offspring developmental outcomes in a parent and grandparent of origin-dependent manner across multiple generations. The first generation of offspring (G_1_) was produced through two reciprocal crosses: cross 1: CC001 females × CC011 males; and cross 2: CC011 females × CC001 males (Fig. 1b). Two sets of cross 1 and cross 2 dams (G_0_) were placed on either CON or LVD diets 5 weeks before mating to generate the first generation of progeny (G_1_). G_1_ offspring were weaned onto normal chow (Teklad 8604), thus minimizing any direct exposure of the pups to the dietary treatments. Cross 1 and cross 2, CON and LVD G_1_ males were next mated to unexposed FVB/NJ (FVB) females to generate the second generation of offspring (G_2_) (Fig. 1b). By outcrossing to unexposed genetically identical FVB/NJ (FVB) dams for G_2_ offspring, we exclude any potential confounding maternal effects such as uterine, X chromosome, or mitochondrial differences.

Vitamin D depletion did not have a significant effect on fecundity (% of matings with litter), fertility (litter size at birth), or offspring postnatal viability (litter size at weaning and male/female ratio) for either generation (Table 1, Additional file 1: Table S1).

**Table 1:** Summary of G_1_ and G_2_ breeding outcomes

	Cross	Diet	No. of litters	Percent of matings with litter (%)	Average litter size ± S.E.M.		Male percentage (mean ± S.E.M.; %)
					At birth	At weaning	
G_1_	1	CON	13	87	7.0 ± 0.6	5.7 ± 0.9	46 ± 6
		LVD	11	83	6.2 ± 0.6	4.8 ± 1.0	51 ± 7
	2	CON	12	75	7.3 ± 0.4	6.9 ± 0.6	51 ± 4
		LVD	9	69	7.1 ± 0.6	7.1 ± 0.6	50 ± 5
G_2_	1	CON	10	91	8.9 ± 0.6	8.9 ± 0.6	53 ± 8
		LVD	7	80	8.4 ± 1.0	8.4 ± 1.0	44 ± 8
	2	CON	7	78	7.7 ± 1.1	7.7 ± 1.1	57 ± 4
		LVD	5	83	9.0 ± 0.5	9.0 ± 0.5	54 ± 6

Litter size at birth does not include litter sizes of zero (0). Litter size at weaning includes litters lost between birth and weaning

*S.E.M.* standard error of the mean

**Fig. 2:**
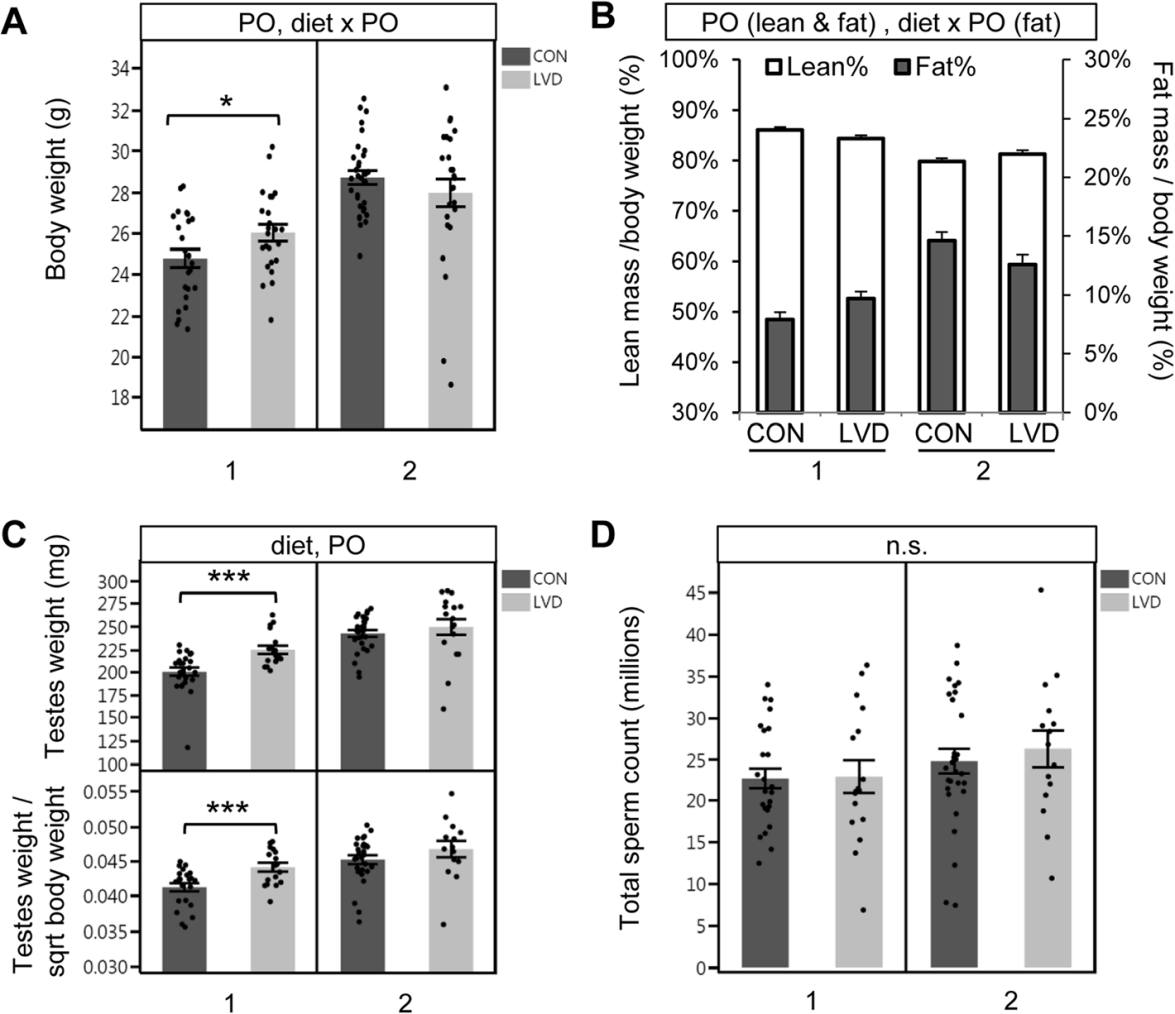
Maternal (G_0_) vitamin D deficiency is linked to (G_1_) offspring developmental outcomes. **a** G_1_ male 8 weeks body weight (*N* = 24, 24, 31, 25). **b** G_1_ male 8 weeks percent lean (left *y*-axis, *open bars*) and fat mass (right *x*-axis, *closed bars*) (*N* = 3, 10, 5, 6). **c** Combined testes weight (*N* = 24, 17, 29, 17, *upper panel*) and testes weight corrected for body weight (*N* = 22, 17, 29, 14, *bottom panel*). **d** Total sperm count (*N* = 25, 17, 29, 15). Each *dot* represents an individual sample. *Error bars* represent standard error of the mean. *Asterisks* (*) or (***) indicate *p* value <0.05 or 0.005 determined by *t* test within each *cross*; *panel above each graph* lists all statistically significant (*p* value <0.05) comparisons determined by regression analysis for overall diet-dependent effects (diet), diet-independent parent of origin effects (PO), and diet-dependent parent of origin effects (diet × PO). *n.s.* not significant

Effect of maternal vitamin D deficiency on development of G_1_ and G_2_ offspring were evaluated by body weight, body composition (percent fat and lean mass), testes weight, and mature sperm count. For G_1_ adult males, we detected both diet-dependent and diet-independent parent of origin differences in body weight, testes weight, and body composition while sperm counts were unaffected (Fig. 2a–d). Cross 1 G_1_ LVD males had significantly higher body and testes weight compared to controls (*p* = 0.04 and *p* = 0.001, respectively, Fig. 2a, c). Interestingly, cross 2 G_1_ LVD males did not differ in testes weight but exhibited a slight trend in decreased body weight compared to controls resulting in a significant diet-dependent parent of origin effect when data from the two crosses were combined (*p* = 0.027) (Fig. 2a, c). Percent fat mass followed a trend similar to body weight for both cross 1 and cross 2 including a significant diet-dependent parent of origin effect (*p* = 0.032, Fig. 2b). This indicates that differences in adiposity are likely responsible for the G_1_ LVD male body weight differences. Diet independent effects were detected for G_1_ male body weight, body composition, and testes weight such that cross 2 males had significantly higher values for all measurements compared to cross 1 (Fig. 2a–c, Additional file 1: Table S1).

**Fig. 3:**
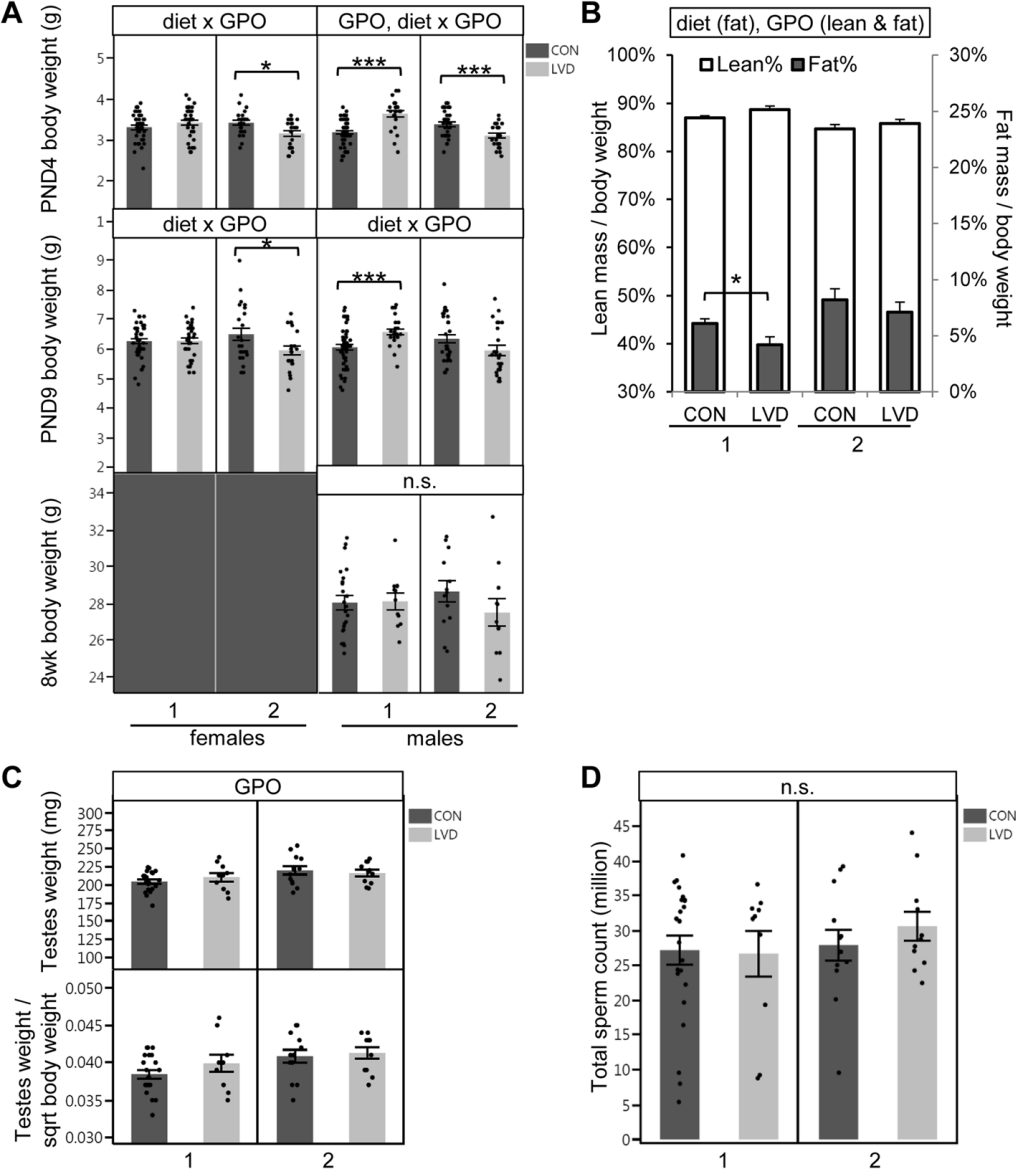
Grandmaternal (G_0_) vitamin D deficiency is linked to G_2_ offspring developmental outcomes. **a** G_2_ male and female PND4, PND9 (female *N* = 40, 35, 23, 21 and male *N* = 49, 24, 31, 24, respectively) and male 8 weeks (*N* = 20, 10, 13, 11) body weight. **b** G_2_ male 8 weeks body composition (*N* = 23, 10, 13, 11). **c** Combined testes weight (*upper panel*) and testes weight corrected for body weight (*bottom panel*) (*N* = 21, 10, 13, 11). **d** Total sperm count (*N* = 23, 10, 13, 11). Each *dot* represents an individual sample. *Error bars* represent standard error of the mean. *Asterisks* (*) or (***) indicate *p* value <0.05 or 0.005 determined by *t* test within each *cross*; *panel above each graph* lists all statistically significant (*p* value *<*0.05*)* comparisons determined by regression analysis for overall diet dependent effects (diet), diet-independent grandparent of origin effects (GPO), and diet-dependent grandparent of origin effects (diet × GPO). *n.s.* not significant

G_2_ developmental outcomes were measured at neonatal stages, postnatal day (PND) 4, PND9, and adulthood (8 weeks). Similar to G_1_ adult males, neonatal G_2_ offspring exhibited diet-dependent and diet-independent grandparent of origin effects on body weight (Fig. 3a). At PND4, cross 1 G_2_ LVD males exhibited significantly higher body weights while cross 2 LVD males exhibited significantly lower body weights compared with controls (*p* = 1.00 × 10^−5^ and *p* = 0.002, respectively, Fig. 3a). G_2_ PND4 females exhibited a similar pattern, with statistically significant differences in cross 2 but not cross 1 (*p* = 0.011 and *p* = 0.201, respectively, Fig. 3a). This body weight pattern was also present at PND9 in both males and females evidenced by a significant diet-dependent grandparent of origin effect detected for both developmental stages (Fig. 3a, Additional file 1: Table S1). The diet-independent grandparent of origin effect on male body weight observed in G_1_ adult males was also detected at PND4 G_2_ males but was not present at PND9 or 8 weeks (*p* = 0.008, *p* = 0.164, and *p* = 0.984, respectively, Fig. 3a).

By adulthood, diet-dependent G_2_ male body weight differences were no longer significant for either cross individually or combined (Additional file 1: Table S1). However, cross 1 G_2_ LVD males had significantly lower percent fat mass compared to controls while cross 2 males had a similar but not statistically significant trend (*p* = 0.034 and *p* = 0.420, respectively Fig. 3b). Furthermore, G_2_ adult males exhibited significant diet-independent grandparent of origin differences in percent fat mass, percent lean mass, and testes weights while sperm counts were seemingly unaffected (*p* = 0.001, *p* = 7.3 × 10^−4^, *p* = 0.029, and *p* = 0.361, respectively, Fig. 3b–d).

Neonatal pup bodyweight is often inversely correlated with litter size since multiparity requires sharing of limited maternal resources. However, we found no significant correlation with diet and litter size (Table 1, Additional file 1: Table S1). Therefore, it is unlikely that diet-dependent body weight effects are confounded by litter size in this study.

### Maternal vitamin D depletion alters DNA methylation in soma and germline of adult G_1_ and G_2_ males in a parent and grandparent of origin-dependent manner

**Fig. 4:**
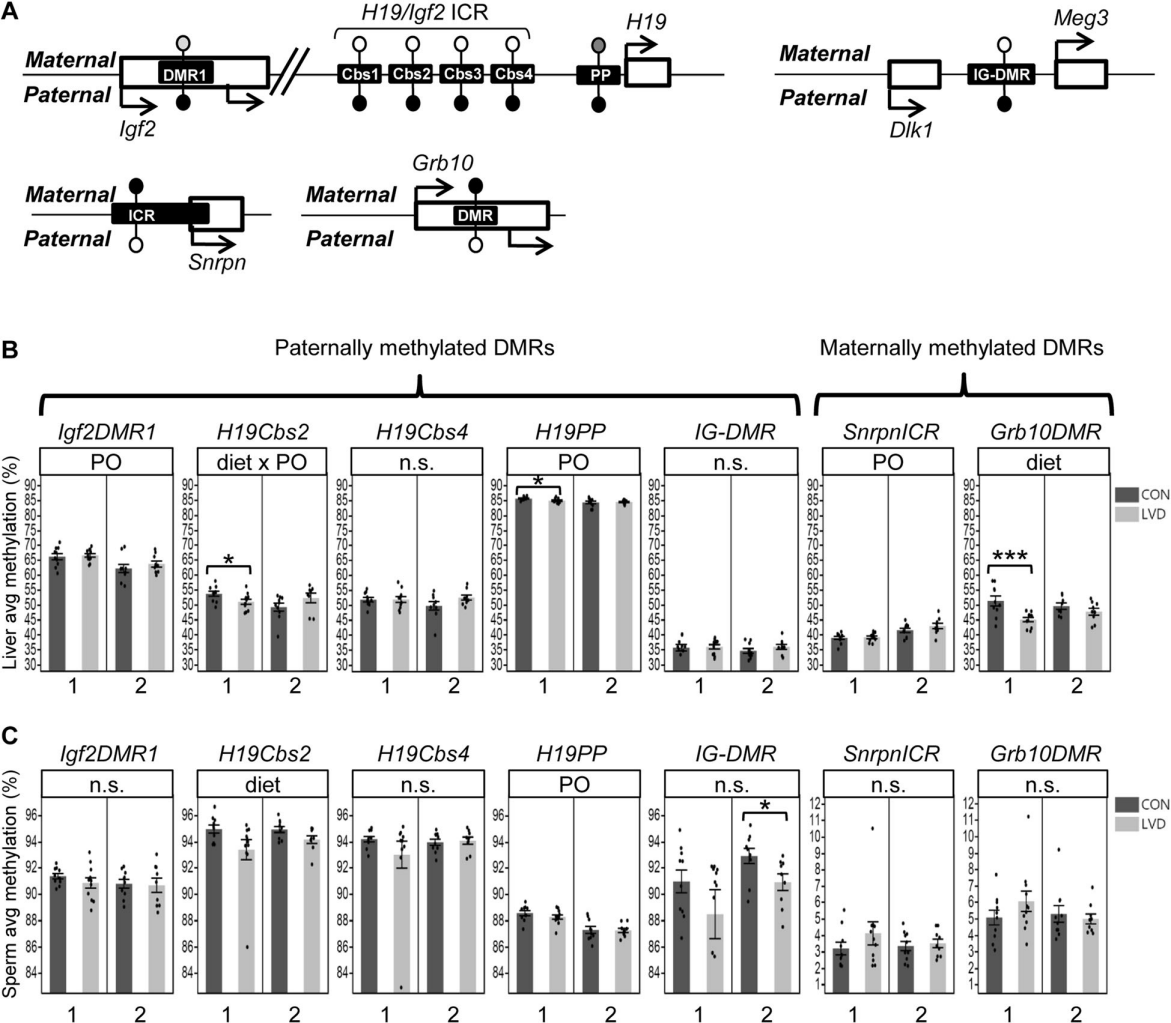
Maternal vitamin D deficiency perturbs DNA methylation at imprinted regions in adult G1 soma (liver) and germline (sperm). **a** Schematic representation of imprinted regions assessed for DNA methylation changes. *White*, *gray*, and *black lollipops* indicate unperturbed methylation states from fully hypomethylated to partially and fully hypermethylated alleles, respectively. Location of lollipop and location of arrows indicates parental methylation or expression from maternal alleles (above gene) and paternal allele (below gene); **b**–**c** *Bar graphs* depict average DNA methylation across samples in the respective treatment group assayed in adult G1 liver (**b)** and sperm (**c)** for each sample analyzed (in order from left to right *N* = 10, 11, 10, 9 for all loci except Grb10 *N* = 10, 10, 9, 8 and *N* = 10, 10, 10, 9 liver and sperm, respectively). For each sample, the average methylation for all CpGs assayed across the locus is represented as a *dot* within the *bar graph* (see the “Methods” section). *DMR* differentially methylated region, *Cbs* CTCF binding site, *ICR* imprinted control region. Median values are indicated and linked by a horizontal line between box and whisker plots. *Asterisks* (*) or (***) indicate *p* value <0.05 or 0.005 determined by *t* test within each *cross*; *panel above each graph* lists all statistically significant (*p* value <0.05) comparisons determined by regression analysis for overall diet-dependent effects (diet), diet-independent parent of origin effects (PO), and diet-dependent parent of origin effects (diet × PO). *n.s.* not significant

We investigated whether vitamin D depletion affects DNA methylation state at imprinted genes in G_1_ and G_2_ males and whether these differences are linked to the developmental changes described above. Adult liver was assayed to determine changes in the soma, and mature sperm was assayed for germline epigenetic effects and to determine transmission potential. DNA methylation states were assayed at imprinted domains regulated by either paternal methylation (*H19/Igf2* and *Dlk1/Meg3*) or maternal methylation (*Snrpn* and *Grb10*) (Fig. 4a) [27]. These imprinted genes play important roles in fetal growth and development [40–44]. Paternally methylated DMRs at imprinted domains are normally hypermethylated in sperm and hypomethylated in the oocyte leading to partial methylation when the two alleles are combined in the resulting somatic tissues of the offspring. In contrast, maternally methylated DMRs at imprinted domains are hypomethylated in sperm and hypermethylated in oocyte but still lead to similar partially methylated loci in offspring soma.

For G_1_ adult liver samples, we found that methylation levels were affected in a diet-dependent and diet-independent manner. Cross 1 LVD liver had significantly lower methylation compared with controls at the *H19/Igf2* ICR (*H19Cbs2*, 2.63 % by mean, *p* = 0.039), *H19PP* (0.68 %, *p* = 0.035), and *Grb10DMR* (6.23 %, *p* = 0.004) (Fig. 4b)*.* Cross 2 liver exhibited a similar but not statistically significant trend for *Grb10DMR* methylation while an inverse trend at *Cbs2* was detected as a significant diet-dependent parent of origin effect (*p* = 0.017, Fig. 4b)*.* Diet-independent parent of origin effects were detected at *Igf2DMR1*, *H19PP*, and *SnrpnICR* (*p* = 0.002, *p* = 0.010, *p* = 3.10 × 10^−5^, Additional file 1: Table S1).

G_1_ adult sperm showed fewer affected loci and smaller methylation changes compared to liver samples. Cross 1 sperm did not exhibit any statistically significant diet-dependent changes; however, cross 2 LVD sperm had significantly lower methylation compared with controls at *IG-DMR* (2.00 %, *p* = 0.030, Fig. 4c)*.* When tested independently neither cross showed significant differences at *H19Cbs2*; however, when the data from both crosses were combined, LVD liver had significantly lower methylation at *H19Cbs2* (1.20 %, *p* = 0.022, Fig. 4c)*.* Interestingly, the diet-independent parent of origin effect on methylation at *H19PP* observed in G_1_ liver was also present in G_1_ sperm (*p* = 1.37 × 10^−6^, Fig. 4c)*.*

**Fig. 5:**
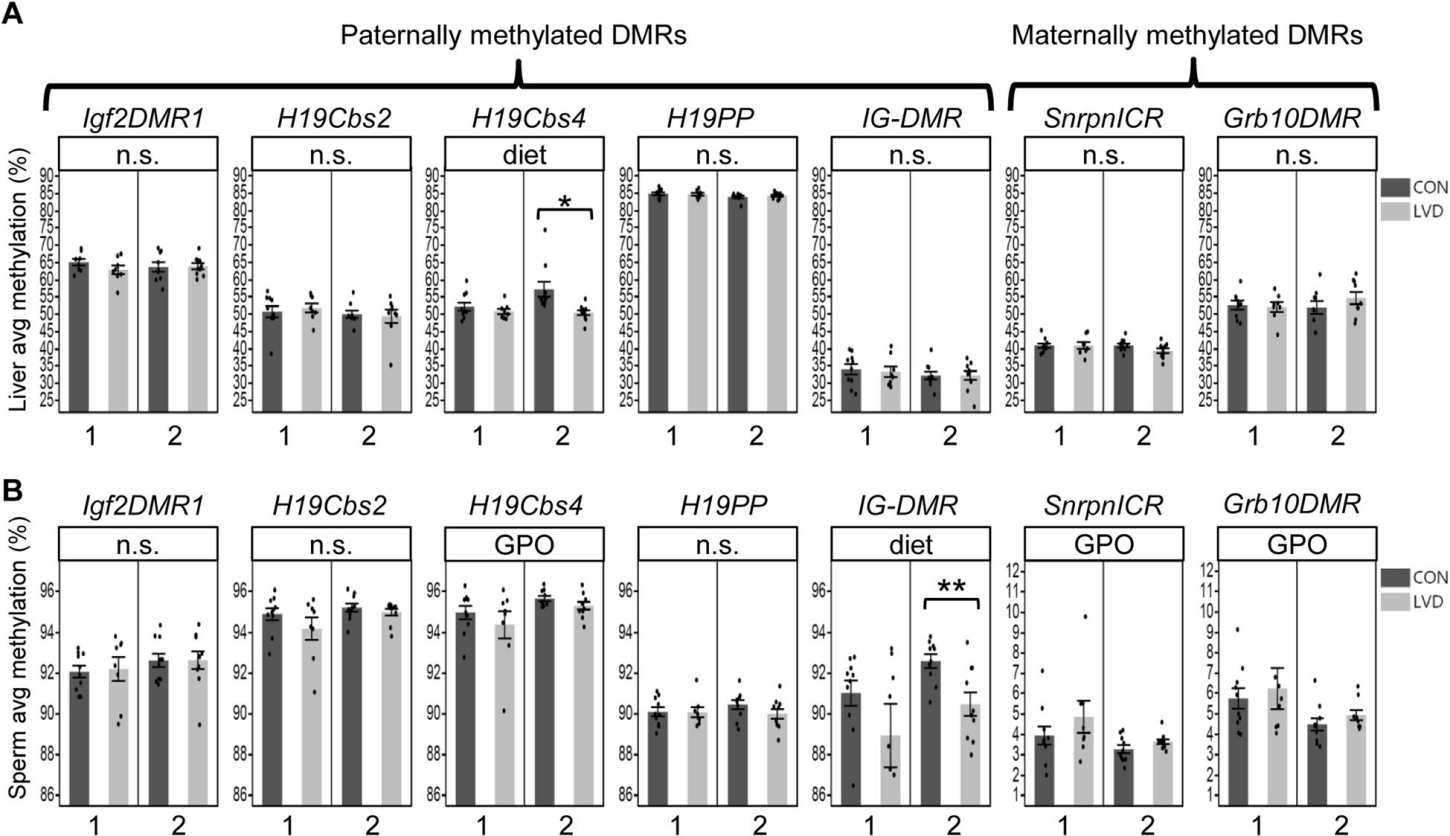
Grandmaternal vitamin D deficiency perturbs DNA methylation at imprinted regions in adult G_2_ male soma (liver) and germline (sperm). **a**, **b** Bar graphs depict average DNA methylation across samples in the respective treatment group assayed in adult G_2_ liver (**a**) and sperm (**b**) for each sample analyzed (in order from *left* to *right*, *N* = 10, 8, 10, 10 for all loci except Grb10 *N* = 10, 7, 9, 9). For each sample, the average methylation for all CpGs assayed across the locus is represented as a *dot* within the bar graph (see the “Methods” section). *DMR* differentially methylated region, *Cbs* CTCF binding site, *ICR* imprinted control region. Median values are indicated and linked by a *horizontal line* between *box* and *whisker plots. Asterisks* (*) or (**) indicate *p* value <0.05 or 0.01 determined by *t* test within each cross; panel above each graph lists all statistically significant (*p* value <0.05) comparisons determined by regression analysis for overall diet-dependent effects (diet), diet-independent grandparent of origin effects (GPO), and diet-dependent grandparent of origin effects (diet × GPO). *n.s.* not significant

To determine whether perturbed methylation states in the G_1_ soma or germline are transmitted to the next generation and to characterize the mode of transmission, we assessed DNA methylation states in G_2_ adult liver and sperm. Overall, we detected fewer loci with significant diet-dependent changes in methylation in G_2_ adult liver compared with G_1_. For G_2_ adult liver, we only detected a significant diet-dependent change in methylation at *Cbs4* in cross 2 or when data from both crosses were combined such that LVD samples had lower methylation levels (6.81 %, *p* = 0.013 and 4.13 %, *p* = 0.006, respectively, Fig. 5a). Interestingly, G_2_ sperm exhibited a change similar to G_1_ sperm such that cross 2 LVD sperm samples had significantly lower methylation levels at *IG-DMR* compared to controls (2.12 %, *p* = 0.007, Fig. 5b). Cross 1 shared a similar trend (although not statistically significant) and when data from both crosses were combined, the effect was significant (*p* = 0.013, Fig. 5b). Diet-independent grandparent of origin effects on methylation were detected in sperm samples at *H19Cbs4*, *SnrpnICR*, and *Grb10DMR* (*p* = 0.027, *p* = 0.033, and *p* = 0.026, respectively, Fig. 5b).

### Vitamin D-dependent epimutations are unstable in the soma and do not reflect transcriptional state

**Fig. 6:**
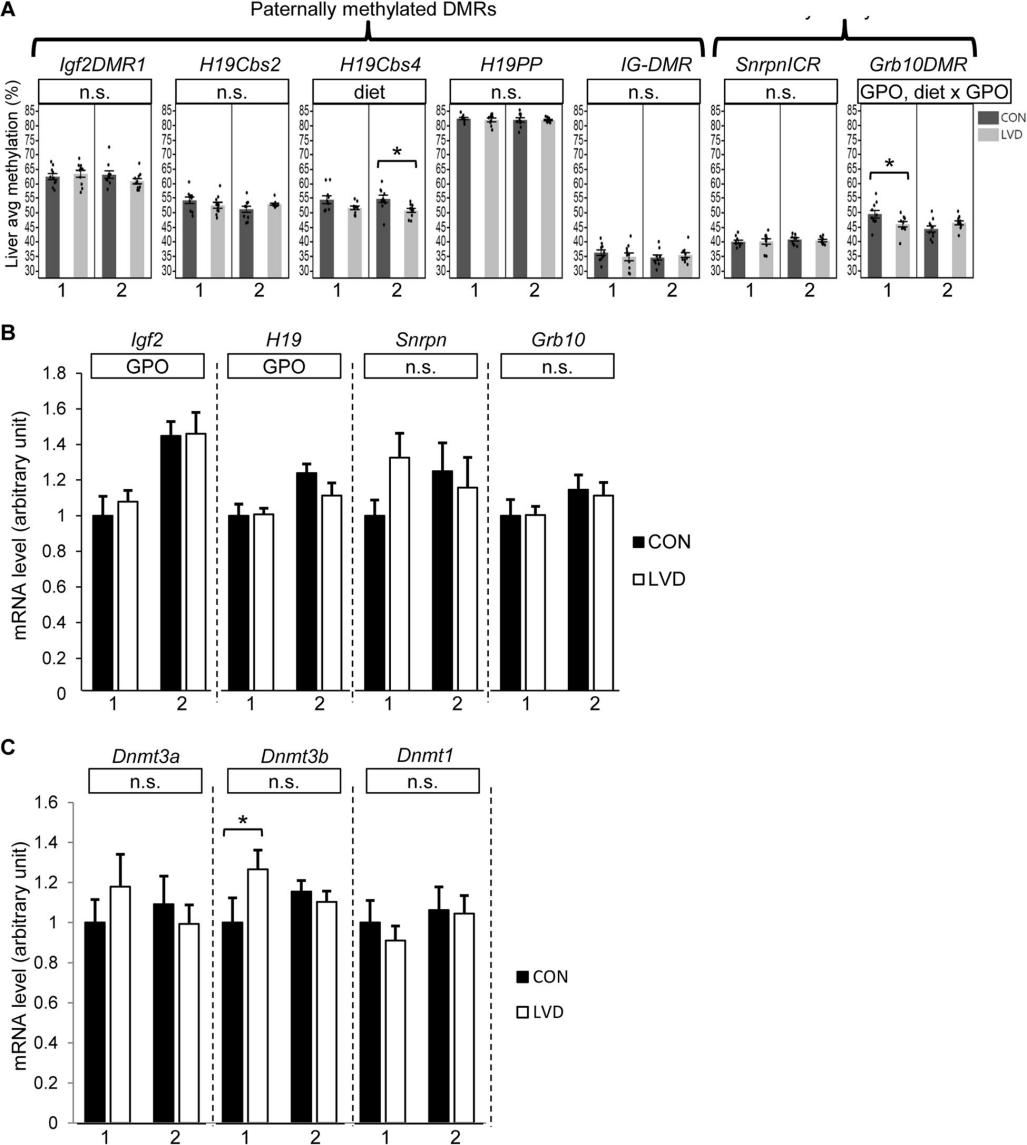
G_2_ male neonatal DNA methylation and gene expression patterns. **a** *Bar graphs* depict average DNA methylation across samples in the respective treatment group assayed in neonatal G_2_ liver (in order from *left* to *right*, *N* = 10 for all loci except *H19PP N* = 10, 9, 10, 10). For each sample, the average methylation for all CpGs assayed across the locus is represented as a *dot* within the bar graph (see the “Methods” section). **b**, **c** mRNA abundance relative to housekeeping genes *Rplp0* and *Gapdh* (*N* = 10 for all genes). *DMR* differentially methylated region, *ICR* imprinted control region. Median values are indicated and linked by a horizontal line between *box* and *whisker plots. Asterisks* (*) indicates *p* value <0.05 determined by *t* test within each cross; *panel above each graph* lists all statistically significant (*p* value *<*0.05) comparisons determined by regression analysis for overall diet-dependent effects (diet), diet-independent grandparent of origin effects (GPO), and diet-dependent grandparent of origin effects (diet × GPO). *n.s.* not significant

To determine the developmental timing of vitamin D-dependent DNA methylation changes in G_2_ adult liver, and thus investigate the mode of inheritance, we assayed DNA methylation levels at the *H19/Igf2*, *Dlk1/Meg3*, *Snrpn*, and *Grb10* domains in G_2_ neonatal (PND9) liver. Similar to G_2_ adult liver, G_2_ neonatal LVD liver samples exhibited significantly lower methylation at *H19Cbs4* compared with controls independently for cross 2 and when data from both crosses were combined (4.00 %, *p* = 0.015, and 3.41 %, *p* = 0.002, Fig. 6a). For cross 1 neonatal LVD liver, methylation levels at *Grb10DMR* were also significantly lower compared with controls (3.46 %, *p* = 0.044, Fig. 6a). This effect at *Grb10DMR* was not present in adult liver (Fig. 5b).

Taken together with methylation changes detected in G_1_ and G_2_ adult samples, these data show that neither neonatal nor adult G_2_ soma findings reflect direct inheritance of LVD-dependent methylation changes in G_1_ sperm. Furthermore, LVD-dependent methylation defects observed in the neonates are not all maintained into adulthood, and methylation defects may emerge in adulthood that are not present at neonatal stages.

To determine whether the vitamin D-dependent epigenetic perturbation reported here results in transcriptional perturbation, we measured relative levels of *Igf2*, *H19*, *Snrpn*, and *Grb10* in G_2_ neonatal liver. There was no significant grandmaternal dietary effect on total mRNA levels for these imprinted genes in G_2_ neonatal liver (Fig. 6b). However, we detected a significant diet-independent grandparent of origin effect for both *H19* and *Igf2* where total expression levels are lower in cross 1 compared with cross 2 (14.6 %, *p* = 0.005 and 28.5 %, *p* = 1.07 × 10^−4^, respectively, Fig. 6b). Overall, these data show that DNA methylation changes at these imprinted loci in G_2_ neonatal liver are not sufficient to significantly perturb total gene expression. Of note, we have previously reported phenotypic changes in the absence of total gene expression changes at the *H19/Igf2* locus but where imprinted expression was perturbed instead [45]. We were unable to assay imprinted monoallelic expression in the G_2_ neonatal liver here since there is no genetic variation between CC001/CC011 and FVB lines at the imprinted genes assayed (see the “Methods” section).

### Vitamin D-dependent methylation changes are correlated with offspring developmental outcomes

The imprinted genes assessed here all play strong roles in pre- and postnatal growth or adiposity [23–26]. Therefore, we determined whether methylation changes were associated with offspring developmental outcomes. *Grb10DMR* exhibited diet-dependent methylation changes in G_1_ adult and G_2_ neonatal livers. Interestingly, for cross 1 G_1_ males, DNA methylation at *Grb10DMR* was negatively correlated with 8 weeks adult body weight (*r* ^2^ = −0.45, *p* = 0.042) and testes weight (before normalization with body weight: *r* ^2^ = −0.52, *p* = 0.015 and after normalization: *r* ^2^ = −0.49, *p* = 0.023). Likewise, DNA methylation at *Grb10DMR* in cross 1 G_2_ neonatal liver was negatively correlated with male G_2_ neonatal body weight (*r* ^2^ = −0.63, *p* = 0.003).

One likely explanation for the sporadic methylation changes observed in response to vitamin D depletion is perturbation of global epigenetic programming as an indirect effect of dysregulation of master epigenetic regulators. Therefore, we assayed relative gene expression of DNA methyltransferases, *Dnmt1*, *Dnmt3a*, and *Dnmt3b* in G_2_ neonatal liver. *Dnmt3a* and *Dnmt3-3b* recognize unmethylated DNA and have important roles in *de novo* methylation during epigenetic reprogramming and pre- and postnatal development. *Dnmt3b* levels were significantly higher (1.3-fold) in the LVD diet group compared with controls in cross 1 (*p* = 0.025, Fig. 6c). Liver expression of *Dnmt3b* in cross 1 G_2_ neonates was positively correlated with PND4 body weight (*r* ^2^ = 0.62, *p* = 0.004), but this correlation was reduced by PND9 (*r* ^2^ = 0.42, *p* = 0.069). Also, *Dnmt3b* expression in G_2_ neonatal liver from cross 1 was negatively correlated with DNA methylation at *Grb10DMR* (*r* ^2^ = 0.42, *p* = 0.069).

## Discussion

These data demonstrate that maternal vitamin D depletion leads to perturbation of DNA methylation at imprinted domains in offspring soma and germline over two successive generations. In both generations, maternal and grandmaternal vitamin D-dependent epigenetic changes coincide with differences in offspring body weight, body composition, and testes weight. The majority of the statistically significant epigenetic effects were relatively small and methylation changes occurred somewhat sporadically, affecting loci differently at each generation in a spatially and temporally dependent manner. We also find that parent and grandparent of origin plays in an important role in the direction and extent of vitamin D-dependent developmental and epigenetic effects in the offspring.

While maternal vitamin D deficiency resulted in both phenotypic and epigenetic changes in both generations, it remains unclear whether the DNA methylation changes are causally linked to the phenotypic outcomes. In support of a potentially causal relationship, we determined a negative correlation between *Grb10DMR* methylation levels and cross 1 body weight in G_1_ and G_2_. This finding does follow the expected relationship since disruption of *Grb10* gene expression is linked to overgrowth [4, 43]. However, in the absence of a significant change in total gene expression, we can only speculate that phenotypic changes may possibly be correlated with loss of imprinted expression. Unfortunately, we lack the ability to measure imprinted (allele specific gene expression) in this study. However, we have shown in previous studies that loss of imprinted expression, even in the absence of changes in total gene expression, are sufficient to perturb phenotypic outcome [45].

This study highlights the importance of maternal vitamin D intake in determining molecular and phenotypic birth outcomes. Although vitamin D deficiency has been linked to adverse pregnancy/birth outcomes [14, 46–52], there is currently no standard practice of screening women for vitamin D deficiency during pregnancy nor are there standard recommendations beyond prenatal vitamins for supplementation during this critical time [53, 54]. Our data highlight two mechanisms that contribute to interindividual differences in response to vitamin D deficiency: the genetic influence on maternal plasma depletion of 25(OH)D and the influence of parent and grandparent of origin in the extent of vitamin D-dependent developmental and epigenetic outcomes.

We demonstrate a genetic influence on maternal 25(OH)D depletion such that CC001 dams on LVD diet had significantly greater depletion of plasma 25(OH)D compared with CC011 dams (Fig. 1c). This is most likely the result of genetic differences in vitamin D metabolism and studies are ongoing to identify and characterize the loci responsible. Further study is also required to determine whether plasma levels of the active vitamin D metabolite 1,25(OH)_2_D are influenced by genetic background. Genetic influences on adiposity may also indirectly explain differences in vitamin D depletion since adiposity has been linked to vitamin D levels [55]. Importantly, maternal strain differences in 25(OH)D depletion are not sufficient to explain all parent and grandparent of origin effects reported here since effects were not unidirectional and often associated with a difference in baseline (control) measurements (e.g., G_1_ body weight, Fig. 2a). We conclude that a more complex mechanism is responsible for the parent and grandparent of origin effects.

Parent of origin-dependent phenotypes are often attributed to epigenetic perturbation of genomic imprinting. Maternal and paternal alleles at imprinted genes are differentially epigenetically marked to regulate imprinted gene expression. Therefore, loss of imprinting on the maternal allele compared to loss of imprinting on the paternal allele often reveals reciprocal phenotypes (e.g., Beckwith Weidemann syndrome and Silver Russell syndrome) [56, 57]. Parent of origin-dependent epigenetic perturbation at imprinted genes has been linked to genetic mutations in *cis* in humans and mouse models [23, 45, 58]. Therefore, the vitamin D-dependent parent of origin effects reported here may be influenced by either epigenetic mechanisms or genetic differences in *cis* or *trans*. The CC001 and CC011 RI strains are inbred and more than 95 % homozygous across the genome [30]. Therefore, G_1_ males for crosses 1 and 2 are mostly genetically identical with the major exception of differences on the X and Y chromosome and mitochondrial DNA. Persistence of epigenetic and phenotypic perturbation in G_2_ males, despite identical maternally inherited X chromosomes and mitochondrial DNA from their FVB dams, excludes the role of X chromosome or mitochondrial DNA in transmission of parent of origin effects. However, we cannot completely rule out the possibility that *cis*-acting allelic differences in G_1_ and G_2_ animals somehow influences the vitamin D-dependent parent of origin epigenetic effects.

**Fig. 7:**
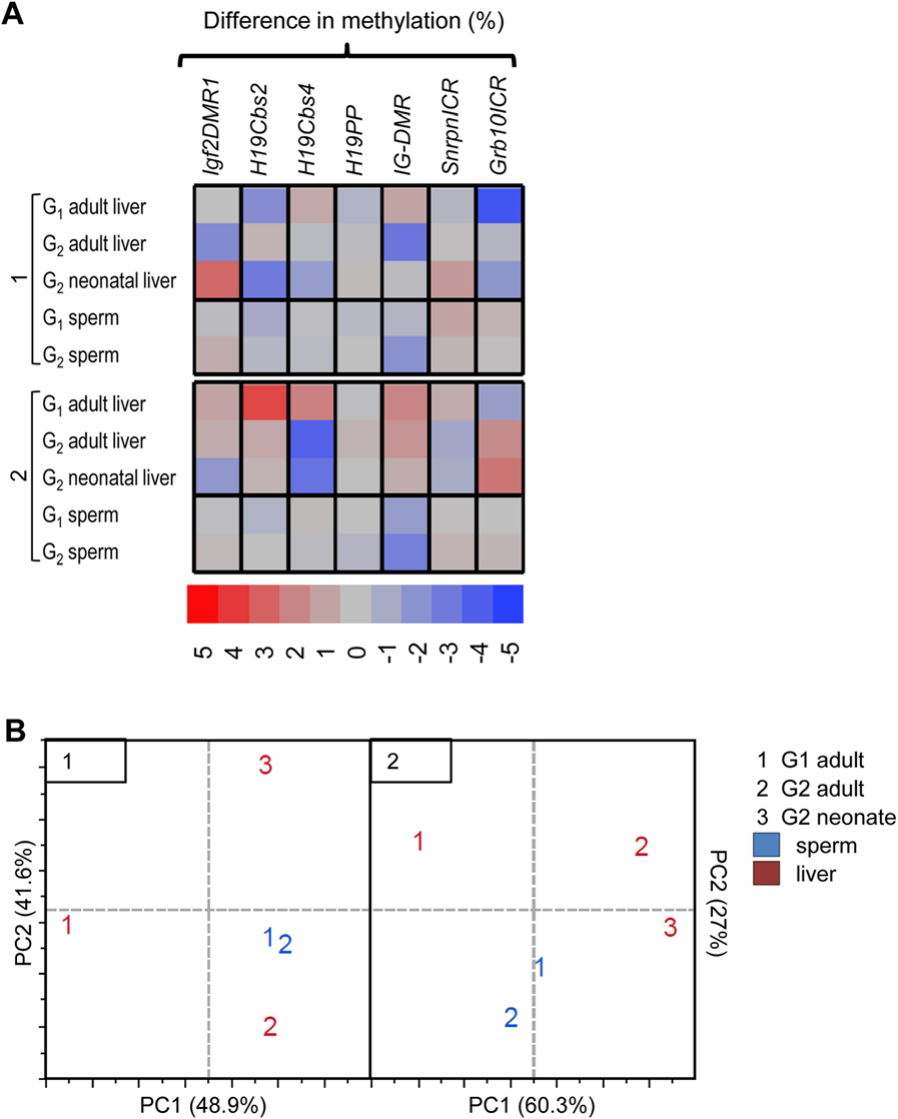
Diet-dependent DNA methylation profile in liver and sperm of G_1_ and G_2_. **a** Heat maps of difference in methylation (value = median of LVD methylation—median of CON methylation) detected in comparing LVD vs. CON groups. *Negative values* indicate hypomethylation in LVD group and positive values indicate hypermethylation in LVD group. Values represented by *shades of color* indicated below each panel. **b** Principal component analysis (PCA) plots for cross 1 and cross 2 was performed for each cell type, generation, and developmental timepoint using difference in methylation at all 7 loci (*Igf2DMR1*, *H19Cbs2*, *H19Cbs4*, *IG-DMR*, *SnrpnICR*, and *Grb10DMR*) between medians of LVD and CON groups. Principal component 1(PC1) and PC2 are the principal components that explain the majority of the variation and are represented on the *x*-axis and *y*-axis, respectively. Percent variation explained by each component is listed in *parentheses* on each axis

The grandmaternal dietary influence on G_2_ phenotype and epigenetic outcome we report here demonstrates inheritance of vitamin D-induced epigenetic perturbation. However, fewer significant changes were detected in the second generation suggesting only partial inheritance. Dietary effects were transmitted solely through the paternal G_1_ lineage since the maternal lineage consisted of genetically identical untreated FVB dams (Fig. 1b). Therefore, we conclude that inheritance is not linked to maternal environment (e.g., uterine effects). Vitamin D-dependent epigenetic perturbation at imprinted loci in the soma and germline of G_1_ and G_2_ offspring did not follow a pattern consistent with direct transmission. This is shown in Fig. 7a, which provides the overall profile of vitamin D-related methylation changes between control and LVD groups for each cell type and generation regardless of statistical significance. This compilation of the data shows little overlap in the loci affected between generations, between cell types, or between developmental stages (neonatal vs. adult) (Fig. 7a). Furthermore, transmission of perturbed DNA methylation profiles did not always follow a pattern consistent with defects in epigenetic reprogramming. For example, we show that the *IG-DMR* is significantly hypomethylated in the LVD group in both G_1_ and G_2_ sperm. However, this locus is unaffected in the neonatal or adult G_2_ soma, which instead exhibits diet-dependent changes at *H19Cbs4* (Fig. 7a). These observations suggest that vitamin D-induced changes in methylation are most likely the result of sporadic epimutations due to global dysregulation of epigenetic maintenance and that disruption of epigenetic regulatory mechanisms is most likely the causal factor. The heritable nature of these effects demonstrates that germline and somatic epigenetic reprogramming are not sufficient to “fix” defects that may have long-term (maintained into adulthood) consequences on epigenetic and phenotypic outcomes.

In support of our hypotheses that maternal vitamin D deficiency leads to global dysregulation of epigenetic maintenance via disruption of epigenetic regulatory factors, we find that *Dnmt3b* transcript levels are upregulated in cross 1 neonatal liver from LVD group compared to CON. These changes are positively correlated with cross 1 neonatal body weight and negatively correlated with cross 1 neonatal *Grb10DMR* methylation. Because *Dnmt3b* plays a primary role in de novo methylation, an increase in expression would cause a direct effect of gain of methylation. Therefore, a negative correlation with *Grb10DMR* indicates an indirect effect.

Our data suggests that vitamin D-dependent dysregulation of epigenetic mechanisms is spatially and temporally regulated. When vitamin D-dependent methylation profiles are compared between cell types, the soma was seemingly more susceptible to perturbation with more loci affected and to a greater extent compared with germ cells (sperm, Fig. 7a). The overall profile of diet-dependent epigenetic changes was more consistent between G_1_ and G_2_ germline while less similar among soma of different generations or developmental stages (Fig. 7a). Principal component analyses (PCA) using the difference in methylation between CON and LVD groups (as shown in Fig. 7a) demonstrate that despite generational differences, germ cell methylation profiles are clustered more closely in both crosses, whereas the soma profiles are more distinct (Fig. 7b). For PC1, the G_1_ soma clusters separately from all other samples (Fig. 7b) indicating that epigenetic changes in the soma resulting from direct exposure to vitamin D deficiency in utero are different than those resulting from indirect exposure in the next generation (G_2_). Furthermore, diet-dependent epigenetic differences between neonatal and adult G_2_ liver samples were cross dependent, being more similar for cross 2 compared with cross 1 (Fig. 7a, b).

## Conclusions

This study expands a growing body of evidence demonstrating that maternal nutrition plays an important role in epigenetic and phenotypic outcome in offspring over multiple generations. Despite our small sample size, many (but not all) of the diet, diet-independent parent/grandparent of origin effects, and diet-dependent parent/grandparent of origin effects maintained an adjusted *p* value <0.10 after correction for multiple testing (Additional file 1: Table S1). Taken together, these data highlight the importance of considering temporal and spatially restricted molecular effects and the influence that parent of origin has on the extent and direction of epigenetic and phenotypic outcomes. We wish to note that this assessment only surveyed methylation at a small number of tightly epigenetically regulated loci and the true biological relevance of these subtle changes remains to be fully determined. However, the seemingly untargeted epigenetic changes at these tightly regulated imprinting regions are indicative of perturbation of epigenetic regulatory pathways that may affect other less tightly regulated loci to a greater extent. Studies are ongoing to characterize genome-wide epigenetic effects of maternal vitamin D depletion including the impact of genetic differences in determining epigenetic and phenotypic outcomes.

## Methods

### Animals—housing, dietary treatment, and breeding scheme

Animal handling was performed in accordance with the *Guide for the Care and Use of Laboratory Animals* under the corresponding animal use protocol at the University of North Carolina at Chapel Hill. Collaborative Cross inbred strains CC001/Unc (CC001) and CC011/Unc (CC011) mice were obtained from the UNC Systems Genetics Core Facility (Chapel Hill, NC) [59]. FVB/NJ (FVB) mice were obtained from The Jackson Laboratory (Bar Harbor, ME). Vivarium temperature was maintained between 21 and 23 °C with a 12-h light cycle. Light source (fluorescent bulbs) was not filtered; thus, all animals were subjected to ultraviolet B exposure (wavelength 280–315 nm) at a rate of 8.39E−7 W per square centimeter. Sterilized water and rodent chow were fed ad libitum.

Figure 1a illustrates all cross and treatment schemes. CC001 and CC011 G_0_ dams were fed either control diet (CON, AIN-93G #110700, 1000 IU/kg of vitamin D_3_, Dyets Inc., PA) or diet lacking vitamin D (LVD, modified AIN-93G diet #119266, 0 IU/kg of vitamin D_3_, Dyets Inc., PA). Dams began this dietary treatment 5 weeks before mating and remained on these diets throughout gestation and weaning. This treatment window ensures depletion of vitamin D before conception and throughout early development of the offspring. All dams in this study were euthanized at pup weaning, and all offspring were transferred at weaning to standard rodent chow (Teklad 8604, Harlan Laboratories).

First generation male offspring (G_1_) were generated from CC crosses by trio or harem breeding (dam X sire, CC001 × CC011 and reciprocal cross CC011 × CC001). G_1_ offspring were weaned at postnatal day (PND) 21 and matured to adulthood. A subset of randomly selected adult G_1_ males was bred to FVB females by trio or harem breeding schemes to generate the second-generation progeny (G_2_). G_2_ birth outcomes and harvests were carried out by the UNC Animal Models Core (Chapel Hill, NC). FVB females were used to maximize fecundity and litter size in G_2_ generation and breeding only experimental G_1_ males eliminated variation caused by strain differences in uterine environment or X chromosome. G_1_ and G_2_ adults were euthanized at 8–9 weeks of age. All G_2_ female neonates and half of all G_2_ male neonates within a litter were euthanized at PND 9 to assess neonatal gene expression and methylation. Tissues and blood were harvested from all animals post euthanasia.

### Quantification of plasma 25(OH)D

Blood was collected by cardiac puncture immediately after euthanizing the animals. Plasma was prepared from heparin-treated blood and snap-frozen in liquid nitrogen. 25(OH)D levels were measured in the UNC NORC Metabolic Molecular Phenotyping Core using ELISA (Eagle biosciences).

### Body weight and body composition measured by magnetic resonance imaging (MRI)

Body weight was measured by balance at 8–9 weeks of age for adults and PND4 and PND9 for neonates. Body composition was measured on 8-week-old males in the UNC Nutrition Obesity Research Center Animal Metabolism Phenotyping Core using MRI (UNC NORC AMP). Free water mass was subtracted from the body weight for calculating fat and lean mass ratio.

### Testis weight, sperm isolation, and counts

Male mice were euthanized at 8 weeks of age by CO_2_ followed by cervical dislocation. Combined testes weight was measured by analytical balance. Intact cauda epididymis and vas deferens were isolated immediately after euthanasia in 1 ml of 37 °Celsius (°C) HTF buffer (pH7.5, 100 mM NaCl, 5 mM KCl, 0.368 mM KH_2_PO_4_, 0.2 mM MgSO_4_, 2 mM CaCl_2_, and 5 mg/ml BSA) and scored under a dissection microscope. Scored epididymis and vas deferens were incubated for 10 min at 37 °C for sperm swim out. Supernatant containing sperm was carefully transferred to 1.5-ml tubes. Sperm resuspension was diluted 1:50 for counting by hemocytometer and remaining cells were pelleted by centrifugation and flash frozen in liquid nitrogen and stored at −80 °C until further analysis.

### DNA methylation measured by pyrosequencing

Liver and sperm samples were harvested immediately after euthanasia and snap-frozen in liquid nitrogen and stored at −80 °C. Genomic DNA was isolated using phenol-chloroform extraction as described previously for DNA methylation assays [60]. Quantity and quality of DNA was assessed by NanoDrop 2000 spectrometer (Thermo Scientific, DE) and spot-checked by the Quant-it PicoGreen dsDNA assay (#P7589, Life technologies, NY). Genomic DNA was bisulfite-converted using the EZ DNA methylation lightning kit (#D5031, Zymo research, CA) according to the manufacturer’s protocol. All pyrosequencing PCR and sequencing primers and sequenced regions were confirmed to be devoid of SNPs or deletions. Pyrosequencing was performed using Pyromark Q96MD instrument (QIAGEN, MD). Loci assayed include previously described DMRs: DMR1 at the insulin-like growth factor 2 gene (*Igf2DMR1*) [45], the *H19/Igf2* ICR (*H19Cbs2*, *H19Rp2* previously) [61], a DMR upstream of the *H19* gene promoter (*H19PP*) [45], a DMR at the intergenic region between the genes delta-like 1 homolog (Drosophila) (*Dlk1*) and maternally expressed 3 (*Meg3*) [62, 63], and a DMR at the small nuclear ribonucleoprotein polypeptide N (*Snrpn*) gene [64]. *H19Cbs4* at the *H19*/*Igf2* ICR was amplified using primers forward 5′-ATGGGTTATTTAGGTATAGTATTTAATGAT-3′ and biotinylated reverse 5′-CTATCCATTTAACTATAACCAAATCT-3′ and pyrosequenced using primer 5′-ATTTTTTGGGTAGTTTTTTTAG-3′. Growth factor receptor-bound protein 10 (*Grb10) DMR* was amplified using primers forward 5′-AGTGAGGTTTAAAAGATGATTAGG-3′ and biotinylated reverse 5′-AAATAAAATTATATAACCATTTTTAATCTC-3′ and pyrosequenced using primer 5′-AATAGTTGTTTATTATTTGGTTGA-3′. PCRs were performed in two replicates and averaged for sequencing. The percent of methylated cytosines at each CpG within the region analyzed was determined by pyrosequencing, and the average methylation of all CpGs combined within the assayed region are presented as avg methylation (%) and used in all downstream analysis. The number of CpGs assayed for each region was 4 (*Igf2DMR1*), 6 (*H19Cbs2*), 5 (*H19Cbs4*), 3 (*H19PP*), 5 (*IG-DMR*), 5 (*SnrpnICR*), and 4 (*Grb10DMR*).

### Quantitative real-time PCR (qRT-PCR)

Total RNA was extracted from liver using Trizol reagent according to the manufacturer’s protocol (#155960926, Life technologies, NY). RNA was DNase treated and reverse transcribed (RT) with negative RT controls (without transcriptase in RT reaction) as previously described [45]; 2.5–5 ng RNA equivalent of cDNA was used for all assays. qRT-PCR was performed using Rotor-gene SYBR green PCR mastermix (#204076, QIAGEN, MD) and Rotor-gene Q instrument (QIAGEN, MD). PCR efficiency for each primer set used was calculated based on standard curves within each run. Expression of target genes was measured relative to both large P0 ribosomal protein (*Rplp0*) and glyceraldehyde-3-phosphate dehydrogenase (*Gapdh*) was calculated using Pfaffl’s method [65]. Target genes, *H19*, *Igf2*, and *Rplp0* (*Arpp0*) were assayed as previously described (HE2 and HE4 for *H19*, Igf2-18 and Igf2-20 for *Igf2*, and GAPDHF1 and GAPDHB1 for *Gapdh*, previously) [58, 66–68]. Primers for *Dnmt1* are forward 5′-AGACCACTGTTCCTCCTTCTA-3′ and reverse 5′-CTCTACCTGGCTCACTACAAAC-3′. Primers for all other targets not mentioned here were purchased from QIAGEN and used according to the manufacturer’s protocol (QuantiTect Primer Assay, QIAGEN, MD; *Snrpn*, catalogue #QT01046486; *Grb10*, catalogue #QT00162813; *Dnmt3a*, catalogue #QT00106519; *Dnmt3b*, catalogue #QT00124264). Samples were amplified in triplicate, and median was used for downstream analysis.

### Assessment of genetic differences between strains

CC founder strain alleles at loci surveyed were determined using the MegaMUGA genotyping array data available through the SGCF website [30]. The presence of SNPs or deletions among CC001, CC011, and FVB strains was determined using the Mouse Phenome Database [69].

### Statistical analysis

Statistical analyses were performed using JMP Pro software version 12.2.0 (SAS, NC). Adjustment for multiple comparisons was performed using R version 3.0.2. Wherever applicable, normality was confirmed by quantile plot and Shapiro-Wilk goodness-of-fit test, and equal variance was tested by Bartlett’s test. Error bars represent standard error of the mean for all bar graphs. Measured variables were compared by either (i) two-tailed *t* test (assuming unequal variance) to determine diet-dependent differences within each cross separately or (ii) regression analyses to determine diet dependent effects that were consistent for both crosses (diet), diet-independent parent/grandparent of origin effects (PO/GPO, cross), and diet-dependent parent/grandparent of origin effects (diet × PO/GPO, diet × cross). For all comparisons, *p* value <0.05 are considered statistically significant. Additional file 1: Table S1 lists all of the comparisons and respective *p* values and description of the statistical models used for each comparison. To demonstrate potential for false discovery, *p* values were adjusted for the number of tests performed within each set of samples using the Benjamini-Hochberg approach [70] and provided as *q* values (Additional file 1: Table S1).

Least squares linear regression was used to determine diet, PO, and diet × PO effects for 25(OH)D levels (with Tukey-Kramer HSD post hoc to determine differences between groups, Fig. 1); litter sizes at birth and litter sizes at weaning (square root transformed) (Table 1); body weight (log10 transformed), testes weight, testes weight/square root of body weight, percent fat mass (fat mass/body weight), percent lean mass (lean mass/body weight), sperm count, DNA methylation levels for *Igf2DMR1*, *H19Cbs2*, *H19Cbs4*, *H19PP*, *IG-DMR*, *Snrpn*ICR, and *Grb10DMR* in sperm, adult liver, and neonatal liver; and gene expression levels for *Igf2*, *H19*, *Snrpn*, *Grb10*, *Dnmta*, *Dnmtb*, and *Dnmt1* (Table 1, Figs. 2, 3, 4, 5 and 6). Generalized linear model with logistic regression of the data as a binomial distribution (JMP, logit link function) was used to determine diet, PO, or GPO and diet × PO or diet × GPO effects for fecundity rates (percent of matings with litter) and percent of males in each litter (Table 1).

Correlations between *Grb10DMR* methylation or Dmnt3b and phenotypic outcomes were calculated by Pearson correlation coefficient. Principal components were calculated based on absolute DNA methylation changes (LVD vs. CON) of the median value for all loci tested for DNA methylation. Two components with the highest eigenvalues were selected for graphing. Heat maps were created showing the difference in methylation calculated from comparing the median methylation changes between LVD and CON diets.

## Additional file

Additional file 1: Table S1. (This additional file contains details for all statistical analysis performed, including *p* values, effect sizes, *q* values, regression model, and method of statistical test.) (XLSX 45 kb)
